# The Triumph of Law

**Published:** 1888-04

**Authors:** 


### THE TRIUMPH OF LAW.

The Indiana State Dental enactment has been fully sustained in a case which was finally adjudicated upon by the Supreme Court of the State. The law is a very good one, its provisions not materially differing from that of some other States. A Board of Examiners is appointed, before whom applicants for registration under the law must appear for the determination of the legality of their diplomas, or for examination as to their qualifications to practice dentistry in the State. Section 3, which was especially applicable in the defendant's case, reads as follows:

> Any person who shall prove to the satisfaction of said Board of Examiners that he is a graduate of a Dental College duly and legally incorporated, and who shall present a diploma therefrom, and shall further show that said college is of good repute, shall be entitled to a registration certificate on the payment of a fee of one dollar to said board.

One George Wilkins, of Marion, Grant County, presented a diploma from the disreputable Delavan, Wis., institution. He was refused registration, but continued practice. Suit was brought against him, and the case was tried in the Circuit Court, the law being held constitutional, and the defendant found guilty and fined. He appealed, and the case was tried before the Supreme Court, a decision being made March 2, 1888. The act was again found constitutional, and the decision of the lower court sustained. Dr. Milton H. Chappell, the Secretary of the Board, has sent us a summary of the opinion of the judges as rendered in the decision, and it is of so much interest to the dentists of all sections, as indicating the reasoning of competent jurists upon the claims of dentistry to legal recognition, that we shall try to present it in another number. It is, much to our regret, crowded out of this one.

